# Breast Carcinoma in Young Females: A Prospective Study in Terms of Clinicopathological Presentation at a Tertiary Care Center in India

**DOI:** 10.7759/cureus.27237

**Published:** 2022-07-25

**Authors:** Mahim Koshariya, Shikha Shukla, Fahad Ansari, Vidhu Khare

**Affiliations:** 1 Department of General Surgery, Gandhi Medical College and Associated Hamidia Hospital, Bhopal, IND

**Keywords:** breast cancer, young females, pr, er, asian women, young women, her2/neu, carcinoma breast

## Abstract

Introduction: The incidence of breast carcinoma in young women is on the rise, particularly in developing Asian countries like India. Owing to a unique presentation in terms of genetic background, clinical features, and histological characteristics, the prognosis becomes challenging, which therefore entails a detailed study for better understanding and management of the disease. This study aimed to establish the role of clinical and pathological parameters in breast cancer disease in young women.

Methods: This was a prospective comparative study conducted at the Department of Surgery, Hamidia Hospital, Bhopal, India, which spanned a total duration of one year between November 2018 and October 2019, and included a total of 98 consecutive in-house breast carcinoma patients. The patients were categorized into two groups based on age, i.e., the young age group (age < 40 years) and the old age group (age ≥ 40 years).

Results: Of the patients, 37 fell in the young age group and 61 in the old age group. There was a significant association between positive family history of breast carcinoma and young age (p = 0.01). Estrogen and progesterone receptor positivity was found to be associated more commonly with old age group patients. The proportion of patients with human epidermal growth factor receptor 2 (HER2)/neu over-expression was higher among the young age group. Triple negativity was more frequently observed amongst young age group patients.

Conclusion: Hormone receptor analysis should be an absolute part of the initial work-up of breast carcinoma. Raising awareness among women in society should be of paramount importance. Family history is crucial, particularly in young women, and should not be dismissed. With timely presentation and effective diagnosis, a safer state with a relatively better prognosis can be achieved.

## Introduction

Breast cancer in young women is a significant healthcare burden. As per previous studies, around 6.6% of all breast cancer cases are diagnosed in women less than 40 years of age [1,2]. Recent global statistics highlight a significant shift over the last few decades in the average age at which a woman gets breast cancer, suggesting that more and more younger women are getting affected [3,4]. Additionally, in many younger patients, the stage of disease at the time of presentation is reportedly advanced and is seen to belong to more aggressive subtypes, e.g., triple-negative or human epidermal growth factor receptor 2 (HER2)-positive breast cancer. With such an early and untoward presentation, it poses a grim challenge to patients, families, and healthcare providers, and thus, necessitates an in-depth analysis and exploration of the subject. This study was conducted at Gandhi Medical College, Hamidia Hospital, to better understand and discern the disease pattern, which would, in turn, help take appropriate measures to prevent, treat, and rehabilitate the patients suffering from it. The chief objectives of the study were to evaluate the patients with breast carcinoma in terms of their age, history, clinical stage, histological grade, and hormone receptors (estrogen receptor (ER), progesterone receptor (PR), and HER2/neu) status, and to compare the data between the two age groups, i.e., old and young.

## Materials and methods

Study design

This was a comparative study conducted at the Department of General Surgery, Gandhi Medical College, Bhopal, a tertiary referral center in India. The study was conducted prospectively, for a period of one year from November 2018 to October 2019, after gaining approval from the ethical committee (Institutional Ethics Committee, Gandhi Medical College, Bhopal; approval no: 36136-38/MC/IEC/2018). The study included all the patients with breast carcinoma who were admitted within the mentioned time frame.

Inclusion and exclusion criteria

All female patients with breast carcinoma admitted to the department were included in the study. Male patients, recurrent breast carcinoma cases, and benign breast diseases later identified as carcinomatous on histopathological examination were excluded from the study.

Data collection

Case records were taken as primary study tools. After obtaining proper consent from the patients, the data were recorded and analyzed on parameters like patients' age, socioeconomic status, family history, tumor size and lymph node status at the time of presentation, clinical stage, pathological grade, and hormone receptors profile (ER, PR, and HER2/neu). The patients were categorized into two groups based on age, i.e., the young age group (age < 40 years) and the old age group (age ≥ 40 years).

Statistical analysis

The statistical analysis was performed using SPSS version 25.0 (IBM Corp., Armonk, NY). The demographic profile was presented using simple descriptive statistics. The categorical data were analyzed using chi-square or Fisher's exact test. The Mann-Whitney U test was used to compare the quantitative data between the two groups. The results were thus analyzed and their mean and standard deviation were obtained. As the level of significance was fixed at 0.05, a p-value of 0.05 was considered to be significant statistically.

## Results

Among the total of 98 patients who were included in the study, 37 patients belonged to the young age group (37.8%) and 61 patients belonged to the old age group (62.2%).

Out of the 37 patients in the young age group, a positive family history of breast carcinoma in first and second-degree relatives was found in four cases (10.8%). In contrast, it was not seen in any of the 61 patients in the old age group (Table 1).

**Table 1:** Family history of breast cancer in study subjects

Family history	<40 years (n = 37)		≥40 years (n = 61)		P-value
	No.	%	No.	%	
Absent	33	89.2	0	0.0	0.01
Present	4	10.8	61	100.0	

**Figure 1:**
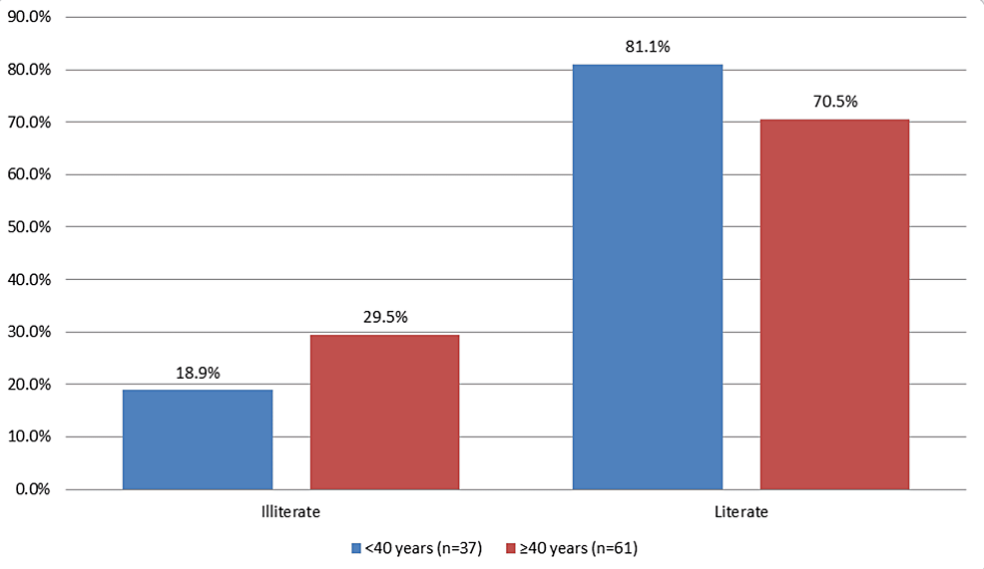
Educational status of study subjects

Among the young age group, out of 37 patients, seven (18.9%) were found to be illiterate, and amongst the older age group, out of a total of 61 patients, 18 (29.5%) were illiterate (Figure 1).

In terms of clinical staging, among the young age group, the majority of cases (51.4%) were found in stage II, while for the old age group, the majority of cases (32.4%) were found in stage III. In both the age groups, stage I presentation was least common (Table 2).

**Table 2:** Clinical stage of breast cancer in study subjects

Clinical stage	<40 years (n = 37)		≥40 years (n = 61)		P-value
	No.	%	No.	%	
I	2	5.4	4	6.6	0.49
II	19	51.4	22	36.1	
III	12	32.4	24	39.3	
IV	4	10.8	11	18.0	

**Figure 2:**
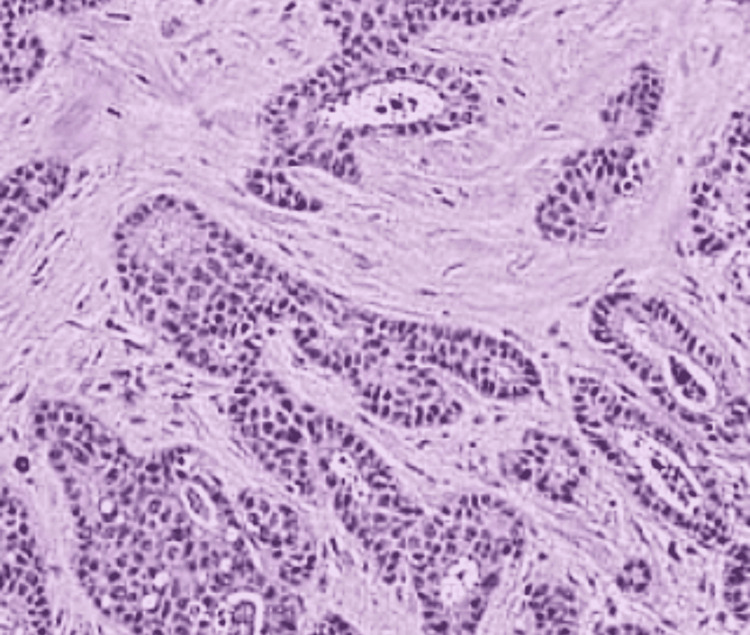
Invasive ductal carcinoma in a 29-year-old female

**Figure 3:**
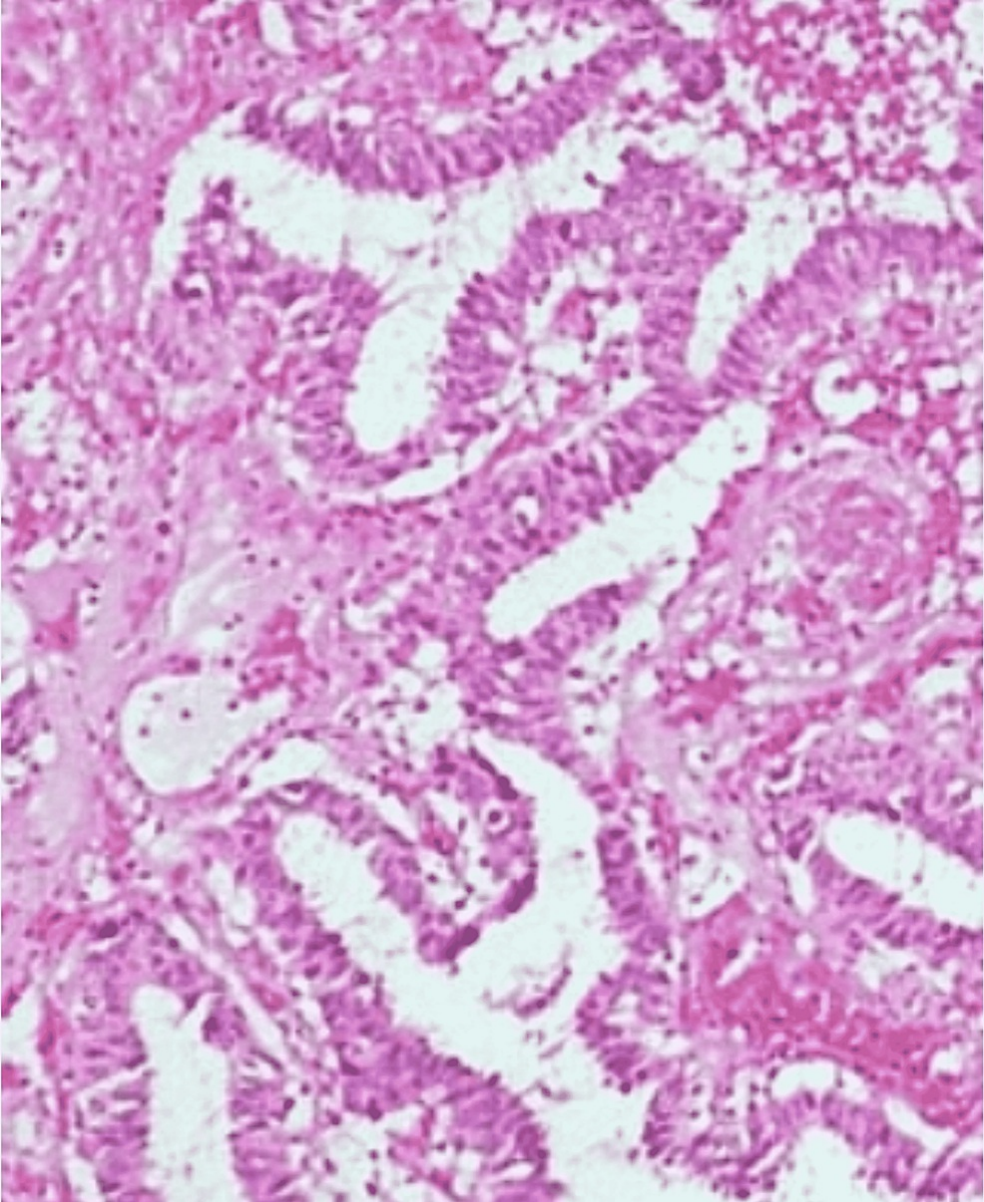
Papillary carcinoma in a 37-year-old female

Histopathologically, the majority of cases in both study groups were invasive ductal carcinoma (IDC) type (89.2% in the young age group and 96.7% in the old age group) (Figure 2). The proportion of papillary was very low (8.1% in the young vs. 3.3% in the old age group) (Figure 3), and invasive lobular was further less (2.7% in the young vs. zero cases in the old age group). The majority of patients in both the study groups (62.2% in the young age group and 59% in the old age group) were found to be exhibiting pathological grade II, followed by grade III (37.8% in young women vs. 27.9% in old women). Grade I was found to be the least common, and there was no presentation in grade I from young age group patients (Table 3).

**Table 3:** Pathological grade of breast cancer in study subjects

Pathological grade	<40 years (n = 37)		≥40 years (n = 61)		P-value
	No.	%	No.	%	
Grade I	0	0.0	8	13.1	0.06
Grade II	23	62.2	36	59.0	
Grade III	14	37.8	17	27.9	

**Figure 4:**
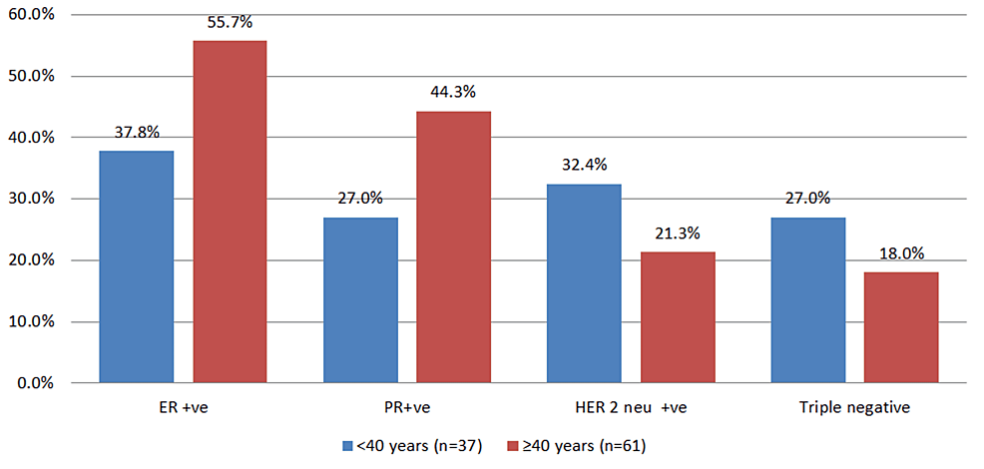
Status of different markers in study subjects ER: estrogen receptor; PR: progesterone receptor; HER2: human epidermal growth factor receptor 2.

In the study, it was also found that the ER and PR positivity was associated more commonly with the old age group patients (ER in 55.7% of cases in the old age group compared to 37.8% in the young age group; PR in 44.3% of cases in the old age group compared to 27% in the young age group). On the other hand, HER2/neu was more frequently seen amongst the young age group patients (32.4% among the young age group compared to 21.3% in the old age group), and triple negativity was also more frequently observed amongst the young age group patients (27% among the young age group compared to 18% among the old age group) (Figure 4).

## Discussion

In developing Asian countries such as India, the impact of breast cancer on young women cannot be disregarded, where the patients are about a decade younger, and the proportion of young patients suffering from breast carcinoma may go up to 25% of the total breast cancer patients [5].

In contrast with older women, young women tend to have more aggressive cancers, and their survival rates are generally lower [6]. The essentials of this fact lie in understanding the basic pathophysiology of human breast development and the idea that breast cancer in young women differs majorly from cancer seen in older women [7]. It has also been observed that the overall incidence of more aggressive breast cancer, which has a higher tendency to metastasize, is on the rise in younger age women [8]. Furthermore, additional issues about young women, e.g., fertility, family planning, body image issues, and financial issues due to the cost of cancer care, need to be addressed.

Various factors such as clinical stage, histologic grade, and hormone receptor status are used to estimate the prognosis. Comparing the two age groups based on these parameters could help delineate the nature of the malignancy, which could further pave the way for better management of this disease.

The association between positive family history of breast carcinoma in young and old age groups was derived, which came out to be statistically significant (p = 0.01). In fact, as per a study by McCredie et al., young women who develop breast cancer are more likely than older women to have an affected first-degree relative [9]. However, in our study, both first and second-degree relatives were taken into account. Additionally, a recall bias may be a possibility that is more likely to be seen among the older age group.

In our study, illiteracy was observed more amongst the old age group patients. Literacy rate can be important, particularly in a developing country like India, and can be employed as a tool to gauge the general awareness among women about their overall well-being and efficiency of healthcare delivery.

In a study conducted by Xiong et al. [10] on 185 young patients, a majority (45%) of patients were found in stage II of the disease, followed by stage III (38%), I, and IV, partly correlating with the findings of this study, where the majority of cases (51.4%) were found in stage II. In fact, as per a study done by Gajdos et al. [11], it was observed that younger age group patients were more likely to be diagnosed with stage II or III cancer (60% vs. 43%, overall p < 0.001). However, in terms of comparison between the two study groups, no significant association could be derived in our study.

In terms of pathological grading, most young women (62.2%) presented with grade II histology. Contrasting findings were present in a study conducted by Nixon et al. [12], where grade III histology in the younger age group was more frequently seen. However, our observation correlates with many previous studies that failed to demonstrate any association of histological grade with age [13-15].

Current guidelines recommend the determination of ER and PR status before planning a definitive therapy for breast carcinoma [16]. In our study, ER and PR positivity was found to be associated more commonly with the old age group; however, the association was not statistically significant. This is similar to studies done by Walker et al. [17] and Hartley et al. [18], which observed a lower incidence of ER and PR positivity in younger women.

HER2 status determination is important as several studies indicate that HER2/neu overexpression is a reliable indicator for low response to tamoxifen and decreased survival [16,19,20].

In our study, the proportion of patients with HER2/neu overexpression was higher among the young age group; however, no significant strength of association could be derived. This is partially similar to a study taken up by Hartley et al. [18], where it was found that younger women had higher levels of HER2/neu overexpression (p = 0.013). However, multiple studies failed to reveal a significant relationship between HER2 overexpression and patients' age [16,21-23].

As per Foulkes et al. [24], triple-negative breast cancers account for 12% to 17% of breast cancers. In our study, triple negativity was more frequently seen amongst the young age group patients (27% among the young age group compared to 18% among the old age group). This is similar to a study done by Carvalho et al. [25], where triple-negative tumors have been seen more frequently in young women, with rates close to 26%. In a study taken up by Collins et al. [26], among 399 women of ≤40 years, 21% were found to be triple-negative. In contrast to this, in a study conducted by Ambroise et al. [27], it was seen that tumors with negative immunohistochemical (IHC) markers (ER, PR, and HER2/neu negativity) were high grade and were mostly seen in the older age group women.

The study attempted to cover many aspects of breast carcinoma from a clinical and histopathological point of view, but certain limitations were incurred. For instance, the sample size was small and could not accurately represent the entire population. The association between age at the time of presentation and family history was significant, but further genetic studies should have been undertaken to establish the role of genetics in younger women. However, owing to a lack of facilities and unaffordability on the part of the majority of patients, it could not be performed. A follow-up to see the response to the treatment could have been beneficial to understand and institute the overall impact of the disease.

## Conclusions

Carcinoma of the breast in young women is a complex disease that is now considered to be a distinct clinical entity. Even though the trend of carcinoma breast cancer is rising, with timely presentation and effective diagnosis, we can achieve a safer state with a relatively better prognosis. Hence, the importance of literacy and self-awareness for young women cannot be undermined. Necessary steps should be taken at the community level to educate women about this issue, the importance of breast self-examination, and the effective utilization of healthcare facilities. Family history is important, particularly for young women, and should not be dismissed while examining patients with breast lesions. Even though the clinicopathological parameters did not vary much between the two age groups, yet the importance of hormone receptor analysis cannot be disregarded and should be taken as a fundamental part of the initial workup of carcinoma of the breast. Screening methods should be made well within the reach of the community. A treatment plan must be designed keeping various issues relating to young women in mind, e.g., infertility, pregnancy, bone health, and psychological well-being. With appropriate and well-organized measures, this burden can be effectively mitigated.
